# Luminescent and paramagnetic properties of nanoparticles shed light on their interactions with proteins

**DOI:** 10.1038/s41598-018-21571-y

**Published:** 2018-02-21

**Authors:** Giuditta Dal Cortivo, Gabriel E. Wagner, Paolo Cortelletti, Krishna Mohan Padmanabha Das, Klaus Zangger, Adolfo Speghini, Daniele Dell’Orco, N. Helge Meyer

**Affiliations:** 1 Department of Neurosciences, Biomedicine and Movement Sciences, Section of Biological Chemistry, Strada le Grazie 8, Verona, Italy; 2 Institute of Hygiene, Microbiology and Environmental Medicine, Medical University of Graz, Neue Stiftingtalstraße 6, 8010 Graz, Austria; 3 Nanomaterials Research Group, Department of Biotechnology, University of Verona and INSTM, UdR Verona, Strada Le Grazie 15, Verona, Italy; 4 Institute of Molecular Biosciences, University of Graz, Humboldtstraße 50/3, 8010 Graz, Austria; 5 Institute of Chemistry, University of Graz, Heinrichstr. 28, 8010 Graz, Austria; 6 Department of Human Medicine and Department of Neuroscience, University of Oldenburg, Carl-von-Ossietzky-Str. 9-11, 26131 Oldenburg, Germany

## Abstract

Nanoparticles have been recognized as promising tools for targeted drug-delivery and protein therapeutics. However, the mechanisms of protein-nanoparticle interaction and the dynamics underlying the binding process are poorly understood. Here, we present a general methodology for the characterization of protein-nanoparticle interaction on a molecular level. To this end we combined biophysical techniques including nuclear magnetic resonance (NMR), circular dichroism (CD), resonance energy transfer (RET) and surface plasmon resonance (SPR). Particularly, we analyzed molecular mechanisms and dynamics of the interaction of CaF_2_ nanoparticles with the prototypical calcium sensor calmodulin (CaM). We observed the transient formation of an intermediate encounter complex involving the structural region linking the two domains. Specific interaction of CaM with CaF_2_ NPs is driven by the N-terminal EF-hands, which seem to recognize Ca^2+^ on the surface of the nanoparticle. We conclude that CaF_2_ NP-CaM interaction is fully compatible with potential applications in nanomedicine. Overall, the methods presented in this work can be extended to other systems and may be useful to quantitatively characterize structural and dynamic features of protein-NP interactions with important implications for nanomedicine and nano-biotechnology.

## Introduction

Nanoparticles (NPs) receive increasing attention in biomedical applications and are intensively discussed as promising drug delivery systems in disease treatment^1–3^. Due to their high degree of specificity and minimal side effects they are of particular interest as they can potentially be used with protein therapeutics in complex diseases. However, the mechanisms of protein-nanoparticle interactions are not fully understood.

Recently, it has been shown that CaF_2_ NPs can specifically bind calcium sensor proteins including recoverin^4^, guanylate cyclase-activating protein 1 (GCAP1)^5^ and calmodulin (CaM)^6^. In these latter cases, binding to NPs occurs at physiological concentrations in a fully reversible manner and most importantly, it does not alter secondary or tertiary structure of the protein^6^. The fact that the surface-bound protein remains structurally and functionally intact suggests that CaF_2_ NPs might be considered as specific carriers for calcium sensors including CaM and GCAP1, and that exploiting the high surface-to-volume ratio typical of the nanoscale could constitute a general strategy for protein replacement-therapy in the case of disease-associated mutant proteins^5^.

CaM is a prototypical calcium sensor protein, which is highly conserved and ubiquitous in eukaryotic cells. It comprises four EF-hands (EF1-4), each containing a functional calcium binding motif and arranged in two domains, termed C-terminal and N-terminal lobe. As Ca^2+^ ions act as important second messenger, CaM is involved in many physiological processes including cell motility, proliferation, apoptosis, cytoskeleton remodeling, metabolic homeostasis, ion transport and protein folding^7,8^. Focusing on CaM-dependent biochemical systems could be particularly useful for targeting cell cycle dysregulation and aberrant proliferation in tumor cells^9^. Very recently, point mutations in the gene encoding CaM were found in patients suffering from arrhythmogenic pathologies^10^, moreover, a significant over-expression of CaM in Alzheimer’s disease has been found^11^, thus implying a possible involvement of CaM in protein therapeutics for a broad variety of diseases spanning from genetic pathologies to neurodegenerative cases.

In this work, we aim at unveiling the mechanisms of protein-NP interactions at the molecular level by focusing on a biologically relevant system, namely CaF_2_ NP interacting with human CaM. We have used a comprehensive approach combining high-resolution spectroscopic techniques with lower-resolution, versatile methods. Specifically, we employed nuclear magnetic resonance spectroscopy (NMR) and resonance energy transfer (RET) by exploiting both paramagnetic and luminescence properties of lanthanide-doped CaF_2_ NPs and obtained further insights in the binding process by circular dichroism spectroscopy and surface plasmon resonance (SPR). Our results suggest that kinetics of protein-nanoparticle interaction in this case is fully compatible with realistic nanomedicine applications. The methodology presented here can be extended to other protein-NP interaction systems of biomedical and biotechnological relevance.

## Methods

### Protein preparation

Calmodulin was expressed in *E. coli* BL21(DE3) Rosetta pLsyS over night at 25 °C after induction with 0.5 mM IPTG (at an OD_600_ of 0.8) using a modified pet24a vector (Genscript). The sequence contains an N-terminal 6His-tag followed by a cleavage site for tobacco etch virus (TEV) protease. Bacteria were grown in LB medium or M9 medium supplemented with [^15^N]H_4_Cl and ^13^C-Glucose for the production of unlabeled and uniformly ^15^N, ^13^C-labeled samples, respectively.

Bacterial cells were harvested by centrifugation for 15 min. at 6000 g and resuspended in 20 mM TRIS-HCl pH 8, 100 mM NaCl, 10 mM imidazole, 4 mM 2-Mercaptoethanol. After addition of protease inhibitor cocktail (Sigma Aldrich), DNase I and Lysozyme bacterial cells were lysed by sonication. Subsequent to centrifugation for 45 min at 48.000 g lysis supernatant was applied to a Ni-NTA-Agarose column (2 ml, 5Prime PerfectPro Ni-NTA Agarose, Thermofisher Scientific). The column was then washed three times each with 10 column volumes of a buffer containing 20 mM TRIS-HCl pH8, 100 mM NaCl, 4 mM 2-Mercaptoethanol and additionally either 10 mM imidazole, 500 mM NaCl or 20 mM imidazole. Protein was eluted with 400 mM imidazole in the same buffer. After removal of the His-Tag by cleavage TEV protease the protein sample was charged with 5 mM CaCl_2_ and applied to a Phenyl-Sepharose (PS) column (GE Healthcare). The column was washed with 20 mM TRIS-HCl pH 7.5, 0.5 mM CaCl_2_, 1 mM DTT and eluted with 20 mM TRIS-HCl, 1 mM EGTA, 1 mM DTT. After extensive dialysis against Ca^2+^-free 50 mM (NH_4_)_2_CO_3_ the protein was lyophilized and stored at −20 °C.

For NMR measurements uniformly labeled protein samples were washed twice with NMR buffer (20 mM BisTris-HCl pH 6.5, 50 mM NaCl, 500 μM CaCl_2_, 0.02% NaN_3_) by ultrafiltration (Amicon Ultra-15 Millipore) and concentrated to 1 mM.

### Protein-fluorescent dye conjugation and luminescence titrations

The maleimide-functionalized fluorescent dye Alexa Fluor 532 (Thermofisher) was specifically coupled to human CaM by a thiol/disulfide exchange reaction. The wild type protein lacks Cys residues, therefore threonine in position 26 was mutated into cysteine using standard mutagenesis techniques. The conjugated dye exhibited ${\lambda }_{exc}^{{\max }}=532\,{\rm{nm}}$ and ${\lambda }_{emi}^{{\max }}=554\,{\rm{nm}}$. After dissolving 1 mg of dye in DMSO, the conjugation was performed in 50 mM TRIS-HCl pH 7.2, 150 mM KCl buffer, in the presence of 10-fold excess of dye with respect to the protein. The dye was added to the protein solution dropwise in the dark, in order to prevent quenching. A buffer exchange step using a PD10 desalting column (GE Healthcare) allowed the isolation of the unconjugated dye. Finally, the conjugation efficiency was calculated using Equation 1:

1 $$Efficiency\,( \% )=\frac{{A}_{532dye}}{{\varepsilon }_{Alexa}\,}\ast \frac{M{W}_{CaM}}{[CaM]}\ast 100$$

in which ε_Alexa_ is the dye extinction molar coefficient (78,000 M^−1^cm^−1^), MW_CaM_ is 16,800 g mol^−1^ and [CaM] is expressed in mgmL^−1^. The efficiency of conjugation was 68%.

In order to study the interaction between the conjugated CaM T26C and the CaF_2_ NPs, titration experiments were performed. Twelve additions (8.05 µL each) of a fluorescently-labeled CaM stock solution (31 µM) were done in a dispersion of 0.5 mgmL^−1^ NPs, moving from a protein-free condition to a final concentration of 5.5 µM CaM. Solution volumes ranged between 500 µL and ~600 µL (dilution effect was considered in the final protein concentration calculation). For each addition, a fluorescence emission spectrum (410–750 nm) was collected following excitation at 980 nm.

### NP preparation

Er^3+^, Yb^3+^ codoped CaF_2_ NPs (nominal molar ratios Ca^2+^:Yb^3+^:Er^3+^ = 0.78:0.20:0.02), Y^3+^,Gd^3+^ co-doped CaF_2_ NPs (nominal molar ratios Ca^2+^:Y^3+^:Gd^3+^ = 0.78:0.21:0.01) and Y^3+^ doped CaF_2_ NPs (nominal molar ratios Ca^2+^:Y^3+^ = 0.78:0.22) were prepared using a hydrothermal technique and stoichiometric amounts of metal chlorides^12^. Sodium citrate was used as a capping agent and ammonium fluoride was added as the fluorine precursor. The obtained solution was heat-treated in a Teflon lined autoclave at 190 °C for 6 hours. After the reaction, NPs were precipitated and collected by centrifugation. The NPs are easily dispersible in water or saline solution. Structural and optical characterizations are reported in previous papers^4–6,13^.

### Limited proteolysis

The pattern of proteolytic digestion was first tested on CaM, in the absence or in the presence of Ca^2+^. In the experiments without NPs, 6 µg CaM in 5 mM TRIS-HCl pH 7.5, 150 mM KCl buffer was incubated with 0.3 µM TPCK-trypsin (trypsin:protein ratio 1:60) for a time range varying from 30 min to 120 min, in the presence of 2 mM Ca^2+^ or EGTA. Since 30 min were found to be enough to reach a complete proteolysis in the presence of EGTA and a limited proteolysis pattern in the presence of Ca^2+^, this condition was used in further experiments with NPs. Mixtures of 21 μM CaM in the absence or in the presence of 3.8 mg mL^−1^ NPs (NPs:CaM = 1:139, stoichiometric ratio was estimated as described elsewhere)^4^ were used in these experiments. The protein was dissolved in 5 mM TRIS-HCl pH 7.5, 150 mM KCl buffer and incubated with 240 µM EGTA or Ca^2+^. Protein and protein-NPs mixtures were incubated with 0.35 µM TPCK-trypsin (trypsin:protein ratio 1:60) for 30 min at 25 °C; the reaction was stopped by adding reducing sample buffer and boiling for 5 min. For each experimental condition a parallel sample without trypsin was tested. Proteolytic patterns were visualized by 15% and 12% SDS-PAGE via Comassie blue staining.

### Circular dichroism spectroscopy

Circular dichroism spectra were collected with a Jasco J-710 spectropolarimeter equipped with a Peltier type thermostated cell holder. Near UV spectra were recorded at 25 °C between 250 nm and 320 nm in a 1 cm quartz cuvette, with scan rate set to 50 nm min^−1^, bandwidth of 1 nm and 4 s as integration time. Reported spectra are the mean of 5 accumulations. Spectra of buffer alone were also collected and considered as blank. Protein concentration was 40 μM, and the spectra were collected in the presence and in the absence of 5.7 mg mL^−1^ NPs (ratio NPs:CaM = 1:139), with 3-fold excess of Ca^2+^/EGTA with respect to the calcium binding sites.

Far UV spectra were collected between 200–250 nm in a 0.1 cm quartz cuvette in the presence and in the absence of 1.75 mg mL^−1^ of NPs (protein concentration was 12 μM). Scan rate, bandwidth, accumulations and saturating conditions were the same as for near UV spectra. CaM previously incubated with NPs was tested without additions of Ca^2+^/EGTA unless differently specified.

Thermal denaturation profiles were monitored between 20 °C and 96 °C in the same conditions as for the far-UV experiments. The scan speed and response time were set at 1 °C/min and 4 s respectively, and the circular dichroism variations was followed at 222 nm.

### Nuclear magnetic resonance spectroscopy

All NMR spectra were recorded on 0.1 mM uniformly ^15^N-labeled or ^15^N,^13^C-labeled CaM on a Bruker Avance III 700 MHz spectrometer equipped with a cryogenically cooled TCI probe-head. Spectra were processed with NMRPipe^14^ and analyzed with Sparky (Goddard and Kneller 2004) and CCPNmr^15^. Backbone chemical shift assignments were obtained from CBCA(CO)NH, CBCANH and HNCA^16^.

For chemical shift perturbation analysis and PRE measurements CaF_2_ nanoparticle or diamagnetic CaF_2_:Y^3+^ and paramagnetic CaF_2_:Y^3+^,Gd^3+^ were added to the NMR sample up to a final concentration of 1.4 mg/ml.

### Surface plasmon resonance spectroscopy

Surface Plasmon Resonance (SPR) experiments were performed using a SensiQ Pioneer instrument. CaM was immobilized via amine coupling on a SensiQ COOH5 sensor chip coated by a carboxylated polysaccharide hydrogel spacer. Carboxyl groups were activated with sequential injections of 60 µL of 10 mM H_3_PO_4_, 60 µL HBS (10 mM HEPES pH 7.4, 150 mM KCl, 20 mM MgCl_2_, 2 mM CaCl_2_), 60 µL 10 mM NaOH and 2 × 60 µL HBS. The sensor chip surface was activated with a 7-min injection of a mixture of 10mM N-hydroxysuccinimide (NHS) and N-ethyl-N’-(dimethylaminopropyl)-carbodiimide (EDC) at a flow of 5 µL min^−1^. The lyophilized protein was dissolved in bidistilled water at a concentration of 1 mg mL^−1^. Subsequent injections of CaM, diluted in Na-acetate buffer and H_3_PO_4_ (pH variating between 2.7–3.1), led the immobilization of 2400 RU (corresponding to ~2.4 ng, 1 RU = 1 pg mm^−2^, flow cell volume <40 nL) in flow cell 1 (FC1), 200 RU in FC3 (~0.2 ng) while FC2 was considered as a reference (no protein injections were performed and the surface was activated/deactivated). Finally, the activated carboxy-groups were blocked via injection of 70 µL of ethanolamine hydrochloride-NaOH pH 8.5. During the immobilization steps, HBS was used as running buffer.

The interactions between immobilized CaM and NPs were investigated by: (a) titrating five different NPs concentrations ranging from 0.1 mgmL^−1^ to 0.5 mgmL^−1^ and (b) injecting the same amount of NPs both in the presence and in the absence of saturating CaM concentration. Interaction experiments were performed with a flow rate of 10 µL min^−1^, with 600 s association time, 1800 s dissociation time and a final 180 s injection of 10 mM K-citrate in order to completely remove residual NP-CaM complex before proceeding with the next injection.

Titration data were fitted to a single exponential decay model, as in Equation 2:

2 $$RU=R{U}_{0}\ast {e}^{-{k}^{off}\ast t}$$

### Instrumentation for luminescence measurements

The upconversion spectra were acquired using a 980 diode laser as excitation source. The emission was analyzed using a monochromator (HR460, Jobin Yvon) equipped with a 1200 g/mm grating and a CCD detector (Spectrum One, Jobin Yvon) with a spectral resolution of 0.15 nm.

## Results and Discussion

### Conformational changes and thermal stability of CaF_2_ NP-bound CaM

**Figure 1:**
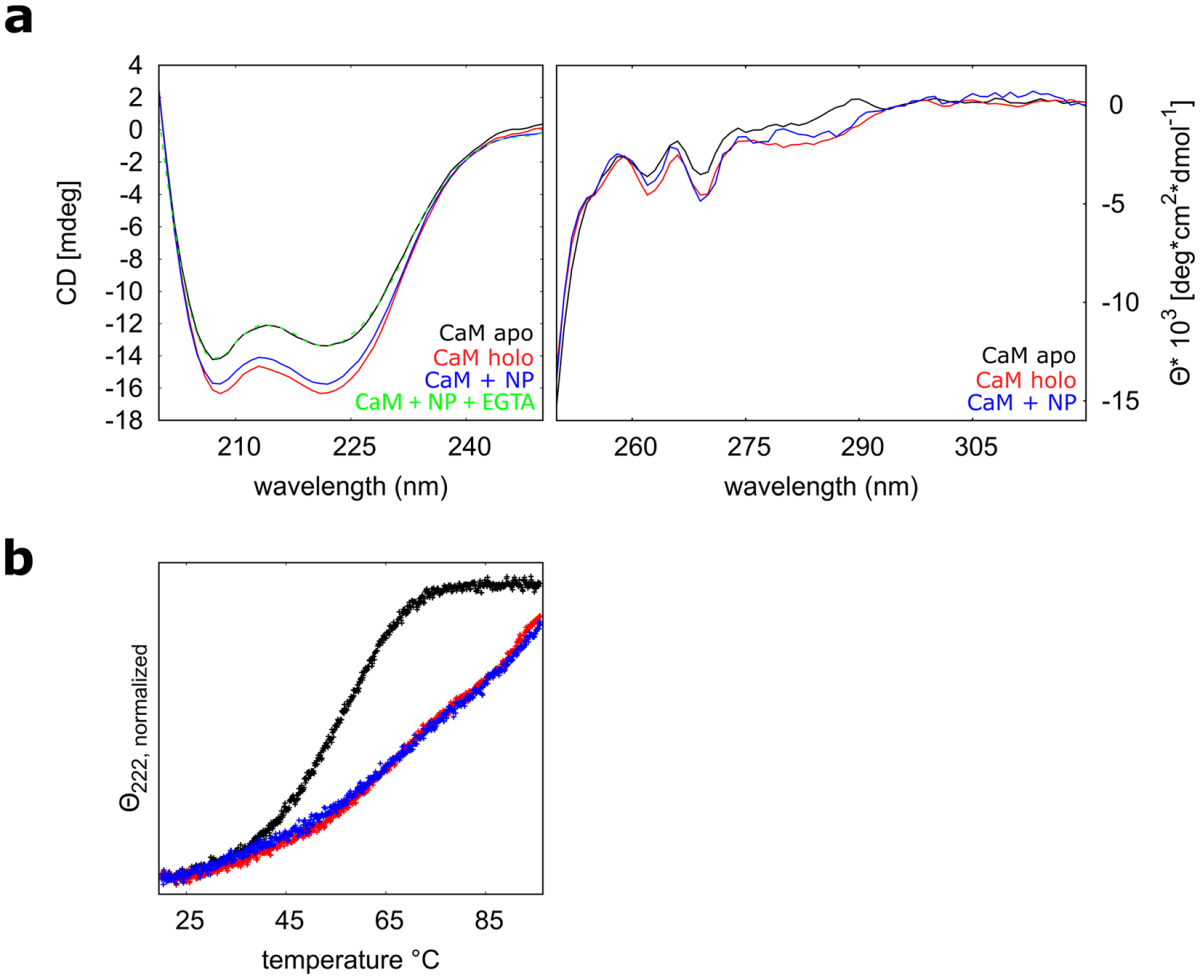
(**a**) Far- and near-UV spectra (of ~12 µM CaM or 30 µM, respectively) in the presence of equal amounts (400 µM) of saturating EGTA (black) or Ca^2+^ (red), and CaM previously incubated with 5.7 mgmL^−1^ NPs (blue). (**b**) Thermal denaturation profiles of ~12 µM CaM previously incubated with 1.7 mgmL^−1^ NPs (blue) and in the presence of saturating (120 µM) EGTA (black) and Ca^2+^ (red). θ_222_ has been normalized to account for different starting values (θ_222, normalized_ = θ_222_/θ_222 at 20 °C_).

We compared conformational changes of CaM upon interaction with Ca^2+^and/or NPs by circular dichroism (CD) spectroscopy, a technique particularly suitable to study proteins in solution under conditions that mimic the physiological ones. While far-UV spectra can reveal secondary structure rearrangements of polypeptides in solution, near-UV spectra provide a fingerprint of their tertiary structure, being sensitive to the micro environment of the aromatic residues. Far-UV CD spectroscopy was used to analyze the secondary structure content of apo CaM (calcium-free, in the presence of EGTA), holo CaM (calcium-bound, in the presence of Ca^2+^) and CaM bound to CaF_2_ NPs. As expected, apo CaM shows characteristics of an alpha helical protein with minima at 208 and 222 nm (Fig. 1a). Decrease of these minima upon addition of Ca^2+^ (21.7% relative change of θ_222_) reflects a major conformational change. (Table 1). A switch in the θ_222_/θ_208_ ratio from 0.95 in the apo form to 1.00 in the holo form has been attributed to an increased alpha helical content, reorganization of the alpha helices and an overall compaction of the protein upon addition of Ca^2+^. These findings are corroborated by NMR structure analysis and SPR^17,18^.

**Table 1:** Far-UV CD data: spectral shapes of apo CaM incubated with CaF_2_ NP are very similar to those of Ca^2+^-loaded unbound protein.

	ϴ_208_	ϴ_222_	ϴ_222_/ϴ_208_	Δϴ_222_/ϴ_222_^a^
CaM Ca^2+^	−16.34	−16.29	1.00	21.7%
CaM EGTA	−14.15	−13.38	0.95	
CaM + NPs	−15.74	−15.75	1.00	17.6%
CaM WT + NPs EGTA	−14.09	−13.39	0.95	

^a^Relative change in ellipticity (θ) at λ = 222 nm as obtained by the difference in θ in the presence of Ca^2+^ or in the apo conditions (EGTA), divided by θ in the apo conditions.

Interestingly, addition of CaF_2_ NPs in a decalcified CaM solution evokes an alteration of the far-UV CD spectrum, which is very similar to the effect of free Ca^2+^ (17.6% relative change of θ_222_) and likewise shifts the θ_222_/θ_208_ ratio to 1.00, suggesting similar rearrangements in the NP - bound form of CaM. If EGTA is added in excess to the CaM-NP solution, the spectral shape shifts back to the apo CaM (Table 1, Fig. 1a), being almost undistinguishable, suggesting a dissociation of the protein-NP complex. The conformational changes of CaM in the presence of either CaF_2_ NPs or free Ca^2+^ also result in a very similar increase in thermal stability, as probed by almost identical thermal denaturation profiles indicating incomplete unfolding at 96 °C, at odds with the complete unfolding observed for the apo form (Fig. 1b).

Changes of the near-UV (250–320 nm) spectra of CaM upon addition of Ca^2+^ or CaF_2_ NPs are indicative of reorganization of the asymmetric environment of aromatic residues which are buried in the core of the protein (Table 1). The near-UV spectra of NP bound CaM are very similar to those of holo CaM, but significantly different from the apo CaM spectrum (Fig. 1a).

**Figure 2:**
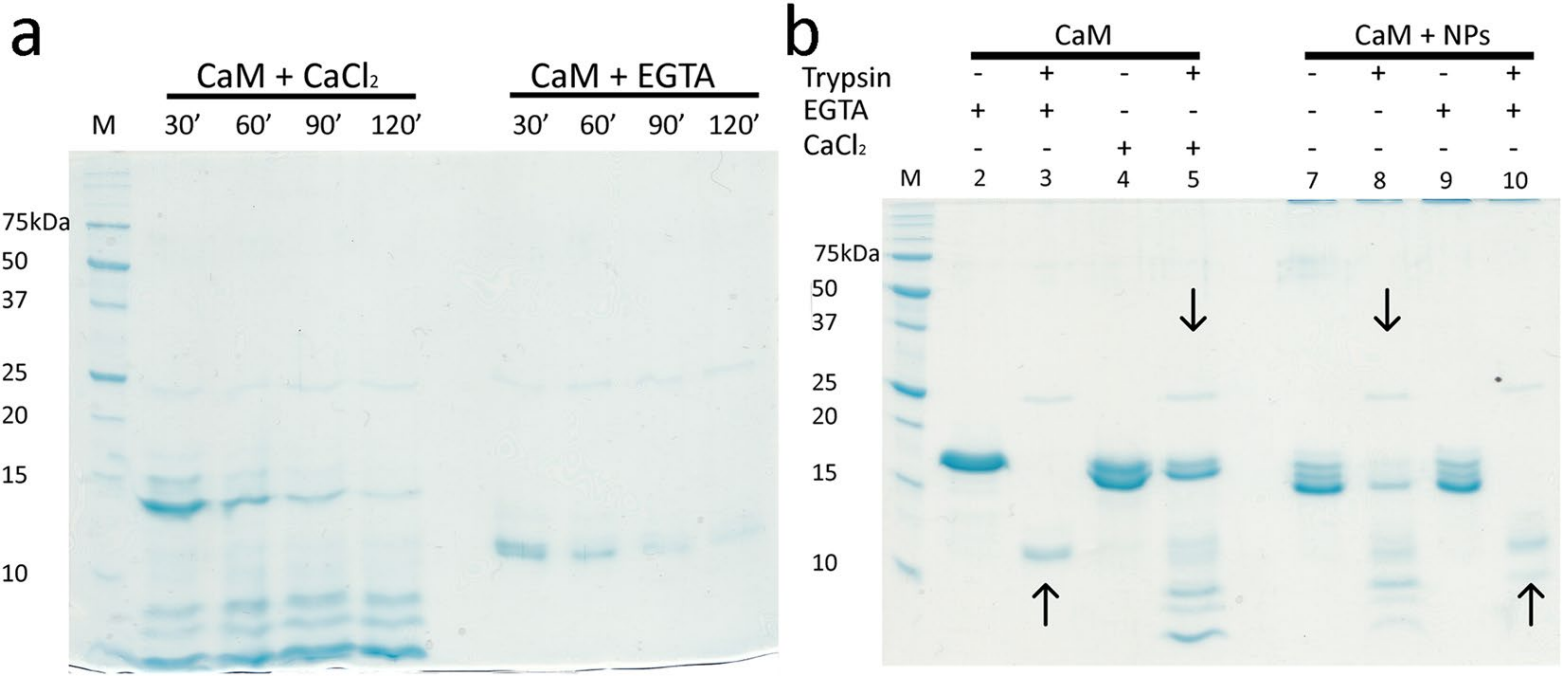
Limited proteolysis pattern of CaM in the presence and in the absence of NPs. (**a**) Proteolysis reactions were performed at 25 °C by 30–120 min incubation with saturating (2 mM) EGTA or Ca^2+^, using a CaM:TPCK-trypsin ratio equal to 1:60. Lane M refers to the protein marker. (**b**) Proteolysis experiments in the presence of NPs were performed by 30 min incubation with saturating (240 µM) EGTA or Ca^2+^, using a CaM:TPCK-trypsin ratio equal to 1:60. Lanes 3 and 5 refer to the digested apo (lane 3) and Ca^2+^-bound (lane 5) CaM. Lanes 8 and 10 refer to the digested CaM previously incubated with NPs (lane 8) and after the addition of EGTA (lane 10). Undigested CaM was loaded in the same conditions in lanes 2, 4, 7 and 9. The similar effect exerted by either free Ca^2+^ or NPs and excess EGTA on the proteolytic patterns is highlighted by downwards and upwards arrows, respectively. The figure results from two separate gels, which have been reported in full-length in Supplementary Figure S1.

To further investigate potential similarities between the Ca^2+^-bound and NP-bound forms of CaM we performed limited proteolysis experiments. Results are reported in Fig. 2.

The presence of free Ca^2+^ clearly protects CaM from complete degradation (Fig. 2a) even after 120 min incubation with trypsin. The apo CaM instead undergoes complete degradation after 30 min. We therefore decided to focus on 30 min incubation for assessing the effects of CaF_2_ NPs on the proteolytic pattern. In line with the spectroscopic results obtained by CD, the presence of CaF_2_ NPs induces a very similar proteolysis pattern compared to the isolated protein in the presence of Ca^2+^ (Fig. 2b, downwards arrows), while excess of EGTA does not prevent complete digestion even in the presence of NPs (Fig. 2b, upwards arrows).

Overall, we thus conclude that the NP-bound conformation of CaM resembles the Ca^2+^ bound state of the protein (Fig. 1c). Strikingly, mutational studies performed with a CaM orthologue from *A. Thaliana* suggested that binding to CaF_2_ NPs requires the presence of intact EF-hand motifs^6^.

### CaM-NP interaction occurs via a two-step process

**Figure 3:**
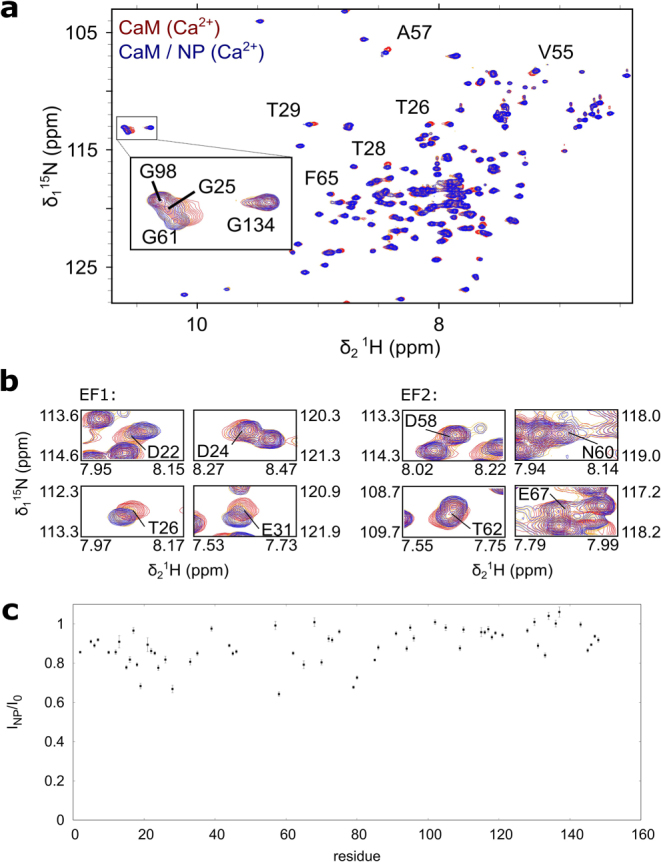
(**a**) Overlay of ^1^H,^15^N HSQC spectra of 1 mM holo CaM (red, 500 μM Ca^2+^) and NP-bound CaM (blue, 500 μM Ca^2+^, 1.4 mg/mL CaF_2_ NP). Residues within the four EF-hands are highlighted. Down-field shifts of the amide protons of G25, G61, G98 and G134 are indicative of Ca^2+^ complexation. Mainly residues close to the N-terminal and not the C-terminal EF-hands experience CSP upon addition of CaF_2_ nanoparticles. (**b**) Close-up of residues which coordinate Ca^2+^ in the N-terminal EF-hand motifs (EF1 and EF2). (**c**) Signal attenuation upon addition of NPs. The ratio of intensity in the presence (I_NP_) and absence (I_0_) of nanoparticles is plotted against the sequence of Calmodulin.

In order to characterize the interaction of CaM with NPs on a molecular level, we used nuclear magnetic resonance spectroscopy (Fig. 3). In particular, we recorded a series of ^1^H, ^15^N heteronuclear single quantum correlation (HSQC) spectra of holo CaM with increasing concentrations of CaF_2_ NPs. This type of spectrum resolves every amide group of the protein as a single 2D peak.

**Figure 4:**
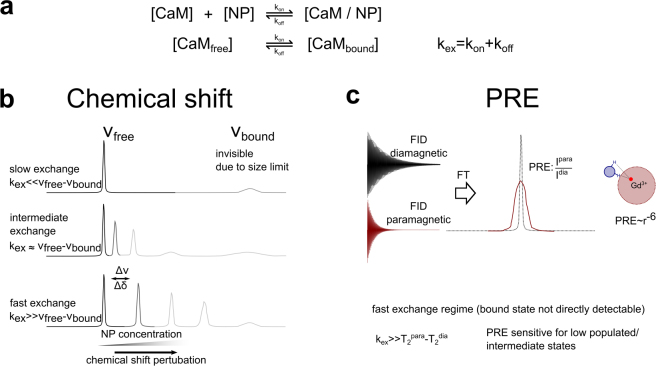
Schematic representation of the association and dissociation equilibria between CaM and CaF_2_ NP as monitored by chemical shift perturbation (CSP) and paramagnetic relaxation enhancement (PRE). (**a**) Chemical equilibrium of CaM/CaF_2_ NP interaction. Exchange between free and NP-bound CaM is determined by the exchange rate constant (k_ex_) which depends on the association and dissociation rate constants (k_on_ and k_off_, respectively). (**b**) CSP upon CaM/NP interaction. Under slow exchange condition NP-bound CaM cannot be observed by NMR. However, given that chemical exchange is fast on the chemical shift timescale, one signal with a population-weighted chemical shift is observable. In intermediate exchange, peaks experience exchange broadening in addition to CSP. (**c**) PRE upon addition of paramagnetic NPs. Interaction with paramagnetic CaF_2_:Y^3+^,Gd^3+^ NPs causes signal broadening. PRE, measured as I^para^/I^dia^, is distance dependent^33^.

Moreover, it is sensitive to changes in the chemical environment of individual amino acids, thus providing a fingerprint of the conformational state of the protein. In a titration experiment residues, which are directly or indirectly involved in the binding interface, typically experience a chemical shift perturbation (CSP) - a change in the resonance frequency of the respective nuclei. CSP has been previously utilized to characterize the binding interface of protein-NP interactions^19–25^. Note that the resonances of the bound state are broadened beyond detection, due to the high molecular weight of the complex. However, molecular details on the interaction can be derived under certain kinetic conditions, i.e. if the exchange between free and NP-bound CaM is fast with regard to the chemical shift timescale (Fig. 4a,b). In this case, a single peak with population averaged chemical shift is visible^19^. If the interaction is in the so called intermediate exchange regime CSPs can be observed as well. However, in this case the observed chemical shift does not represent a simple population weighted average of the bound and the free state. Additionally, exchange broadening of the respective peaks occurs.

In order to achieve intermediate to fast exchange condition 500 μM Ca^2+^ was added to the NMR sample, which allows a rapid exchange between Ca^2+^-bound and NP-bound CaM, as no conformational rearrangement is required. Under these conditions CaM seems to bind preferably to NP, even in the presence of high [Ca^2+^] as significant CSPs are observed upon addition of NPs (Fig. 3a,b).

**Figure 5:**
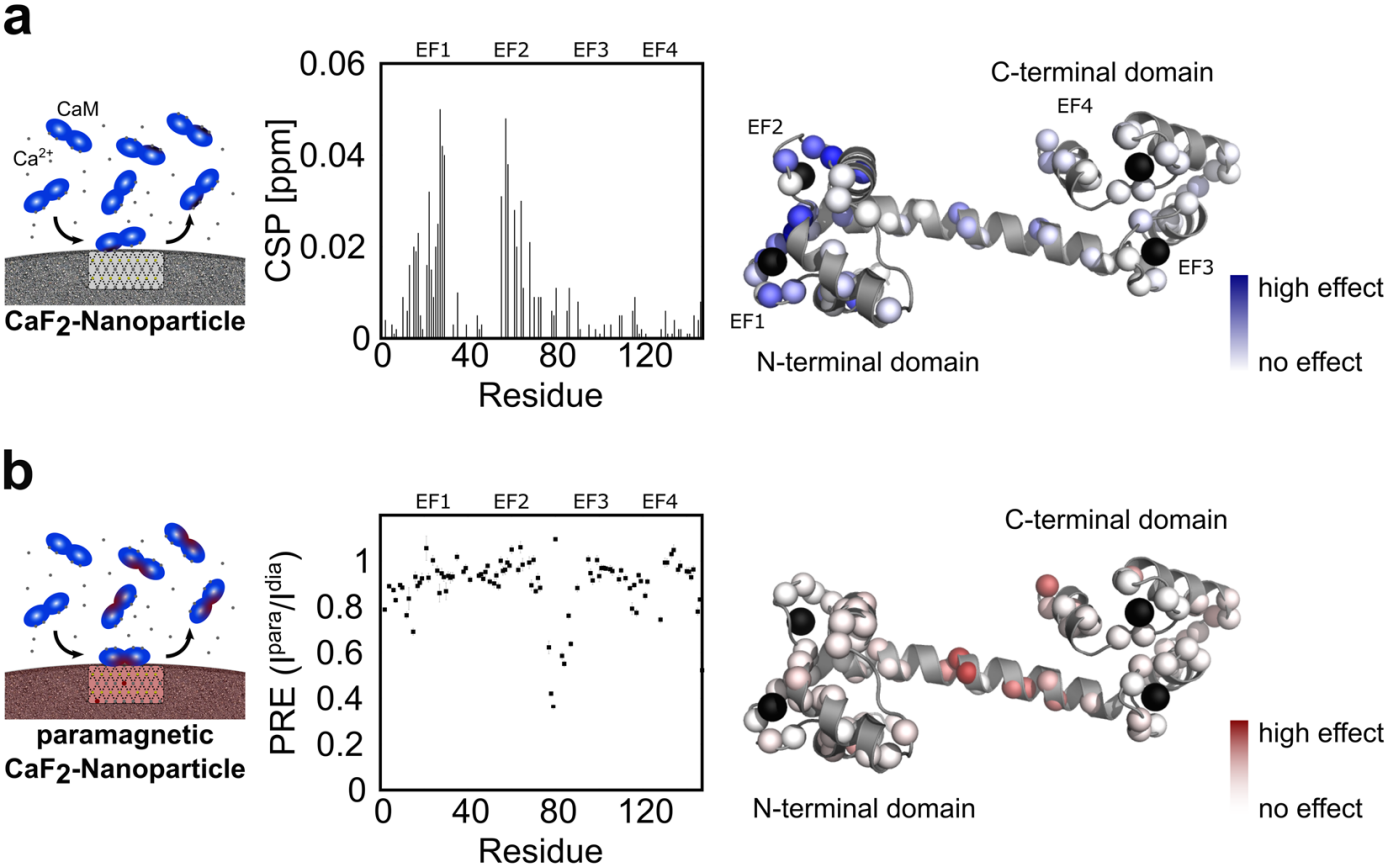
(**a**) Chemical shift perturbation (CSP) and (**b**) paramagnetic relaxation enhancement (PRE) upon CaM/NP interaction is plotted against the sequence of CaM and visualized on the protein structure. Position of the four EF-hands is indicated as EF1-4.

This can be attributed to the high local concentration of Ca^2+^ on the surface of the NPs. Intriguingly, the strongest CSPs are observed for residues of the two N-terminal EF-hand motifs (EF1 and EF2) closely located near the respective calcium binding sites (Fig. 5a). EF3 and 4 in the C-terminal lobe of CaM experience only little CSP. In addition, amino acids 80–87 - the central segment of the helix, which is connecting the N- and C-terminal lobe of CaM – is also affected upon binding to NPs.

Assuming that the NMR signal is a population-weighted average of holo CaM and NP-bound CaM an attenuation of the NMR signals upon addition of NPs is expected. Indeed, we observe an overall reduction of signal intensity. The signal reduction is more pronounced in those regions that also experience CSP, namely the N-terminal lobe and the linker region. The C-terminal domain, however, shows slightly less pronounced signal reduction. We thus conclude that the C-terminal domain is not in direct contact with the NP and thus showing dynamical behavior which is significantly different from the NP in terms of molecular tumbling. The average intensity ratio I_NP_/I_0_ in the N-terminal domain is 0.84 ± 0.08 with the intensity in presence and absence of NPs, I_NP_ and I_0,_ respectively, thus 16% of CaM is bound to NP under these conditions. As the average CSP on CaM NP interaction is rather small, we conclude that the conformations of holo CaM and NP-bound CaM are very similar.

Paramagnetic relaxation enhancement (PRE) is based on a dramatic effect of unpaired electrons on NMR spectra and has been widely used to characterize dark states, e.g. transiently populated states or high molecular weight complexes, by NMR spectroscopy^26,27^. Recently, PRE has been used to characterize the interface of ubiquitin transiently absorbed to lanthanide-doped, paramagnetic SrF_2_:Y^3+^, Gd^3+^ NPs^24^. We adapted this approach to study the interaction of CaM with CaF_2_ NPs. For our experiment we used paramagnetic CaF_2_:Y^3+^, Gd^3+^ NPs and CaF_2_:Y^3+^ NPs as the diamagnetic reference. Figure 5b shows the PRE, described as the signal intensity of the paramagnetic state over the intensity in the diamagnetic state (I^para^/I^dia^), plotted against the sequence of CaM. Residues which experience the highest PRE fall in a region between amino acids 77 to 87, representing the centre of the interconnecting helix, congruent with the region identified by CSP. However, residues involved in calcium binding of all four EF hands show only very little PRE. This observation is consistent with the occurrence of a short-lived encounter complex, which is most likely driven by an unspecific electrostatic interaction between a negatively charged region on CaM (the sequence of the linker stretch is very acidic ^77^MKDTDSEEE^87^) and Ca^2+^ on the surface of the nanoparticle.

PREs are population-averaged with 1/r^6^, where r is the distance between the paramagnetic center and the nucleus under investigation. Therefore, even short-lived intermediates, which bind close to the paramagnetic center can yield significant PREs, although CSPs can be small or even unobservable for such transient interactions^28^. On the other hand, PREs can only be observed when the complex is in fast exchange with an exchange rate k_ex_ >10^2^ s^−1 ^^26^. This is likely the reason for the small PREs observed in EF hands 1 and 2. Thus, the binding event can best be described by a two-step process. A transient encounter complex in fast exchange is formed between the central helical region of CaM with the NP as indicated by PREs (Fig. 5b). This fleeting interaction is in equilibrium with a tighter complex between EF1 and EF2 with calcium sites on the nanoparticle as evidenced by CSPs (Fig. 5a).

### Affinity and kinetics of CaM-NP interactions are physiologically relevant

In order to directly study the interaction of apo CaM and NP we utilized Resonance Energy Transfer (RET). An important feature of the CaF_2_ host is that it can be suitably doped with luminescent lanthanide ions. In particular, Er^3+^,Yb^3+^ doped CaF_2_ NPs can generate strong upconversion (UC) emission upon excitation in the near infrared (NIR) region by using a cheap 980 nm diode laser^29,30^. UC emission generated by the Er^3+^/Yb^3+^-doped CaF_2_ NPs upon NIR excitation (donor) was used to excite the suitably labelled protein (acceptor). A single point mutation in between the two N-terminal EF-hands, namely T26C, was introduced in CaM and used to site-specifically label the protein with a fluorophore (Alexa Fluor 532) that is excited at 530 nm and emits fluorescence at a maximum wavelength of 554 nm.

**Figure 6:**
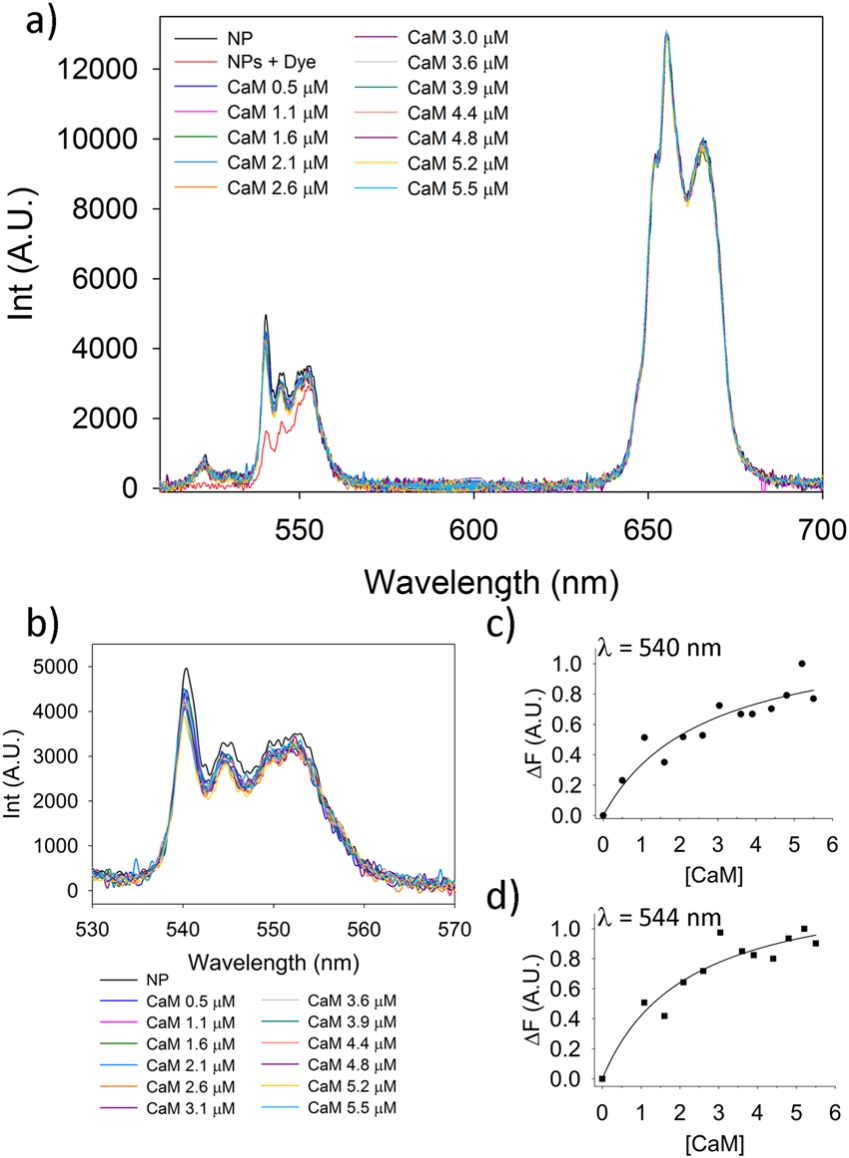
RET analysis of CaM-NP interaction (**a**) Upconversion spectra of CaF_2_ NPs in CaM titration experiments. The resonance energy transfer (RET) phenomenon is evident by a strong decrease of the Er^3+^ emission in the 520–560 nm optical range. Band assignment: (i) ^2^H_1/2_→^4^I_15/2_; (ii) ^4^S_3/2_→^4^I_15/2_; (iii) ^4^F_9/2_→^4^I_15/2_. (**b**–**d**) Example of titration experiments of Alexa Fluor 532-conjugated CaM with a 0.5 mg/mL NPs dispersion. (**b**) Zoom of the spectral area showing the RET phenomenon. Notice the change in NPs emission at 540 (**c**) and 544nm (**d**).

Indeed, a clear RET signal can be detected upon the interaction of CaM with NPs. Furthermore, titration experiments show a concentration-dependent RET phenomenon, as demonstrated by the decrease in intensity of emission bands at 540 and 544 nm, with conserved spectral intensity in the 650/670 nm range, compatible with an apparent K_D_ of 2.5 μM for CaM-NP binding. (Fig. 6) This is in line with previous data obtained by ITC with orthologue CaM (K_D_ = 2 μM) from *A. Thaliana*^6^.

**Figure 7:**
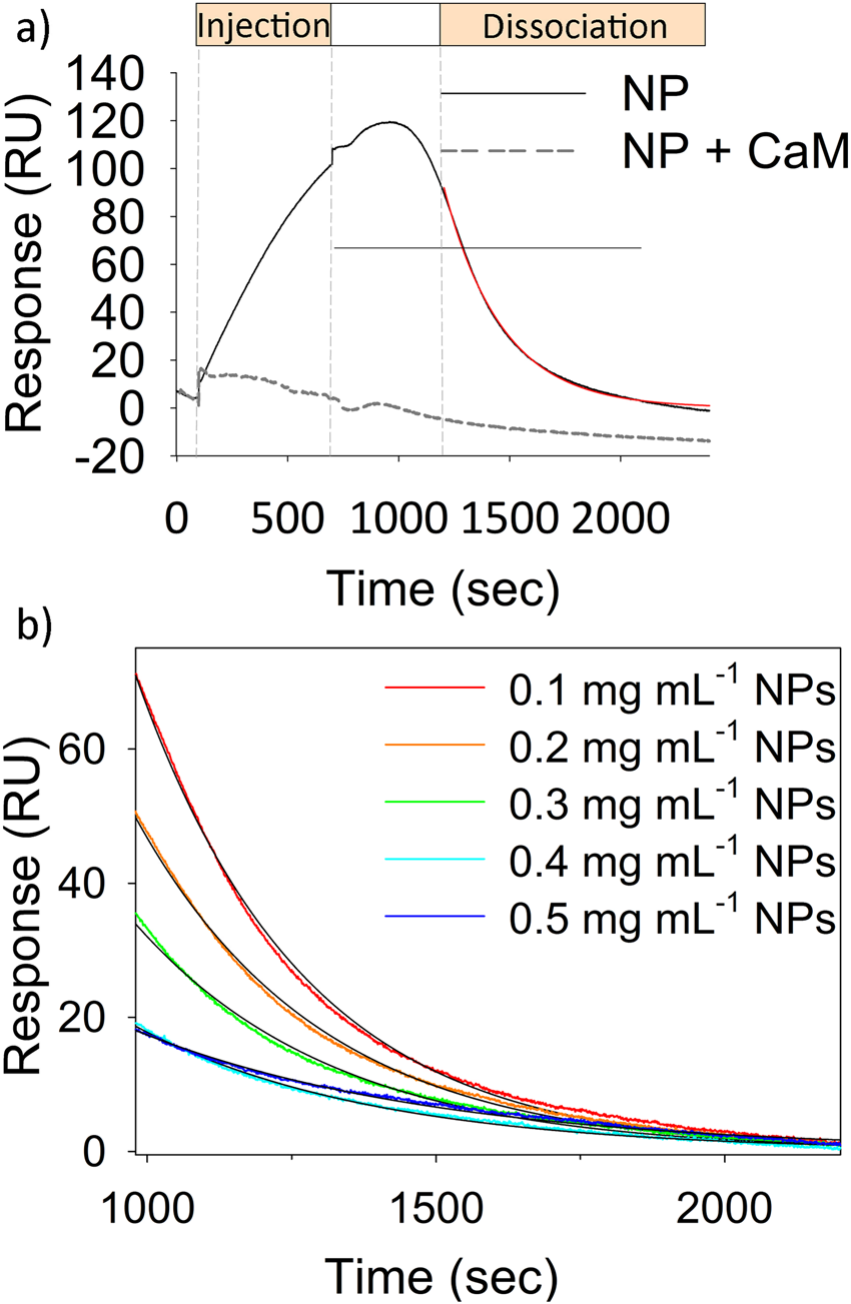
SPR analysis of CaM-NP interaction. (**a**) Example of sensorgram obtained by flowing NPs (0.1 mg/mL) on a flow cell, where 2400 RU of CaM were previously immobilized via amine coupling. Injection was performed for 600 s (flow rate 10 µL/min) and dissociation was followed for 1800 s. The red curve refers to data fitting using a single exponential function. The dashed grey line refers to injection of the same amount of NPs previously incubated with 1.6 µM CaM. (**b**) Example of dissociation curves obtained by SPR overlapped to the fitting curves according to a single exponential in the 0.1–0.5 mg/mL NP range.

The kinetics of CaM-NP interactions was monitored by SPR. Similarly to what was recently observed with GCAP1^5^, CaF_2_ NPs interact with immobilized CaM if the surface of the particle is uncoupled to other CaM molecules. In the case of a “protein corona” formed by CaM molecules previously incubated with NPs, no association was indeed observed (Fig. 7). The dashed grey line in Fig. 7a reports, as an example, the case of 1.6 µM CaM incubated at room temperature with 0.1 mg mL^−1^ NPs; similar results were obtained with other amounts of NP/protein. Several injections of NPs in a 0.1–0.5 mgmL^−1^ concentration range showed that, similar to the GCAP1 case^5^, the association process does not follow a Langmuir adsorption model, as no specific dependency on the NP concentration could be detected for the SPR signal. Moreover, approximately 400 s are needed for the system to relax after NP injection (Fig. 7a), a phenomenon that was previously interpreted as a combination of protein conformational change^31^,^32^ and NP diffusion in the polysaccharide matrix on the sensor chip^5^ that reflects in a ~200 s continuous increase in the SPR signal after the injection of NPs was interrupted. When real dissociation is concerned, however, the dissociation phase could be nicely fitted to a single exponential function, leading to a k^off^ = (3.5 ± 0.4) × 10^−3^ s^−1^, compatible with a fairly faster dissociation, which appeared to be completed in 2000 s, thus being fully compatible with potential nanomedicine applications. The fact that the complete dissociation for GCAP1 was observed after ~6500 s under similar conditions probably reflects the significantly higher affinity of GCAP1 for CaF_2_ NPs (12 nM)^5^.

## Conclusions

**Figure 8:**
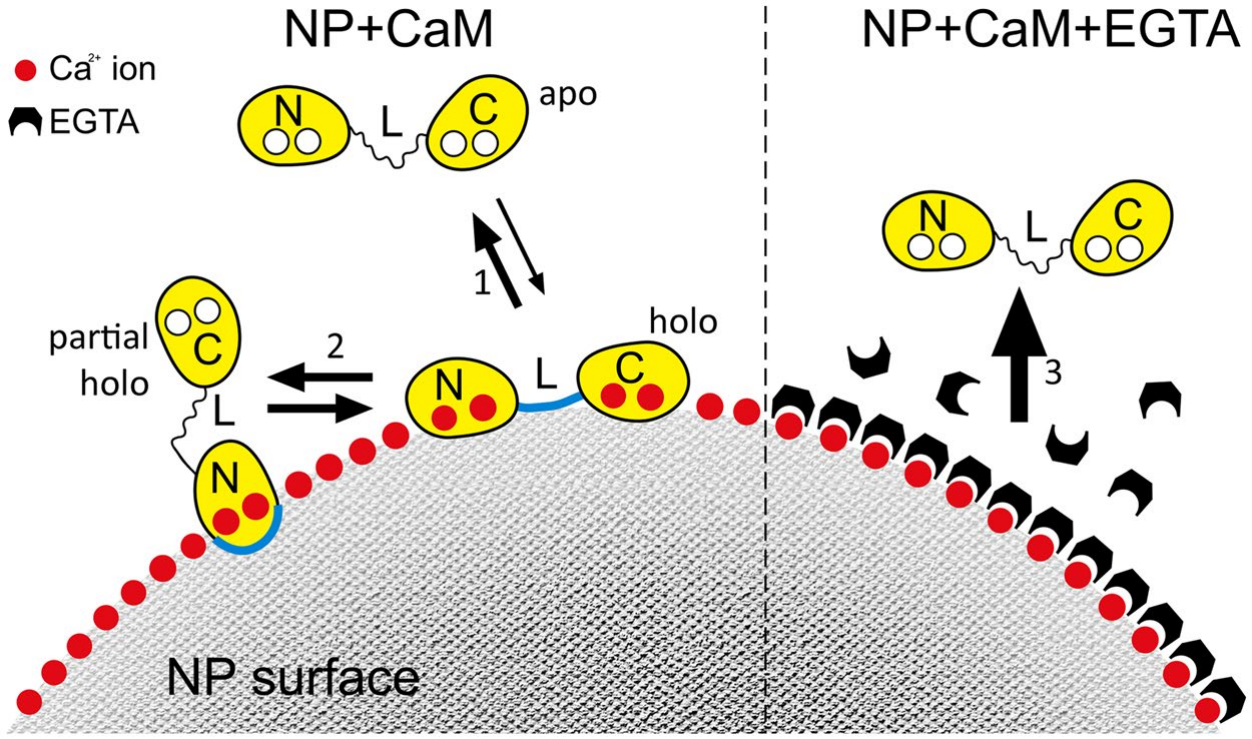
Schematic representation of NP-CaM interactions. Left side: when apo CaM is incubated with NPs, two different equilibria establish. PRE experiments suggest that the first contact between CaM and NP is mediated by the central linker (L) region. At this point, CaM can either return to the apo state (1) through a rapid dissociation by the NP surface, or be converted into a more stable NP-bound conformation (holo), via an interaction mostly involving the N-terminal domain (2), as suggested by CSP experiments. Right side: when an excess of EGTA (black blocks) is added, the chelator binds Ca^2+^ ions on the NP surface, thus restoring the apo-CaM conformation (3), as suggested by near- and farUV spectra (Fig. 1a). Blue thick lines represent the areas mostly involved in CaM/NPs interaction.

In this study, we present a general methodology to comprehensively describe the binding of proteins to NPs by using a combination of biophysical techniques. As a specific example, we found that the binding of the prototypical calcium sensor CaM to CaF_2_ NPs can be described by a two-step process, involving the formation of an intermediate encounter complex involving the linker region. Specific interaction of CaM to CaF_2_ NPs is driven by the N-terminal EF-hands, which seem to recognize Ca^2+^ on the surface of the nanoparticle. If high amounts of chelating molecules such as EGTA are added to the system, the dissociation of CaM from the NP surface is favored and brings the protein back to its apo-form. Interestingly, some of us previously demonstrated that CaM dissociated from the CaF_2_ NP surface is functional and can fully activate a protein target^6^. We summarize the emerging model of interaction between CaM and CaF_2_ NPs in Fig. 8.

A major advantage of our comprehensive analysis is that, beside a static picture of the protein-NP system at the equilibrium that can be obtained by several physicochemical approaches, it offers a more dynamic description that provides information as to the kinetics of the association/dissociation processes. While further studies are needed to thoroughly decipher the complex kinetics of association, we clearly showed that once the association to CaF_2_ NPs has occurred, for instance by previous incubation, the dissociation of CaM from the NP surface is complete in 2000 s, thus pointing to physiological relevance of the process.

The unique feature of having a large surface-to-volume ratio confers NPs the possibility to safely carry a functional protein on the surface of the device. This could be exploited in future studies for delivery purposes in the increasing cases in which CaM has been shown to be associated to specific disease states.

### Availability of materials and data

All materials, data and protocols associated with this manuscript are promptly available to readers without undue qualifications in material transfer agreements.

## Electronic supplementary material

Supplementary Information
