# Supplementary material for: Luminescent and paramagnetic properties of nanoparticles shed light on their interactions with proteins
Source: Sci Rep. 2018 Feb 21;8:3420. doi: 10.1038/s41598-018-21571-y (PMC5821874; doi:10.1038/s41598-018-21571-y)
Supplement: Supplementary file 1 — Supplementary Information [file 41598_2018_21571_MOESM1_ESM.pdf]

Supplementary information

Luminescent and paramagnetic properties of nanoparticles shed light on their interactions with proteins

by Giuditta Dal Cortivo, Gabriel E. Wagner, Paolo Cortelletti, Krishna Mohan Padmanabha Das, Klaus Zangger, Adolfo Speghini, and Daniele Dell'Orco, N. Helge Meyer

Supplementary Figure S1

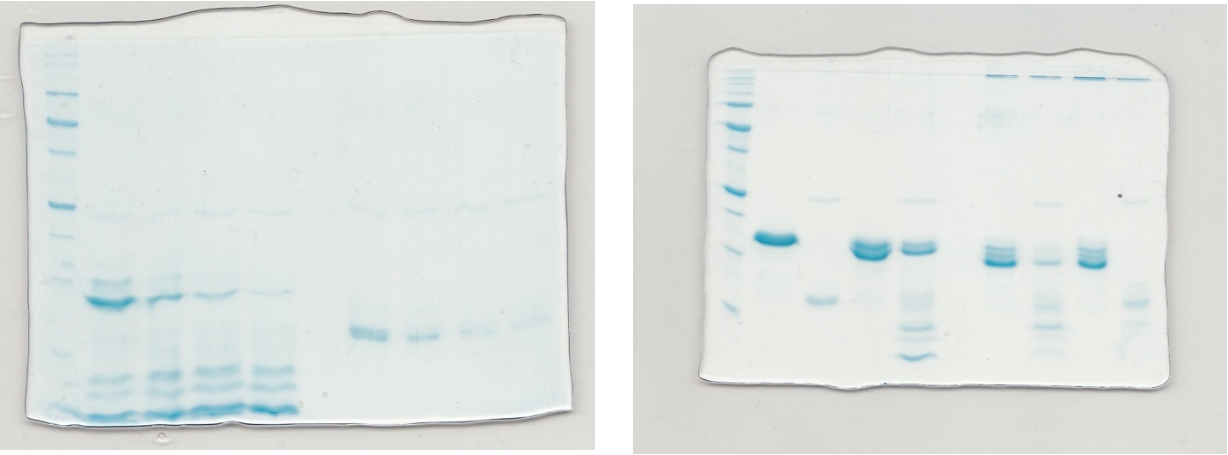

Full-length gels (uncropped) used to prepare panels ***a*** (left) and ***b*** (right) in Figure 2, respectively.
